# Vaccination coverage, delay and loss to follow-up of the triple viral vaccine, in live births between 2017 and 2018 in Brazilian cities

**DOI:** 10.1590/S2237-96222024v33e20231218.especial2.en

**Published:** 2025-01-10

**Authors:** Tatiana Lang D’Agostini, Fernanda Florencia Fregnan Zambom, José Cássio de Moraes, Ana Paula França, Jéssica Pires de Camargo, Manoel Carlos Sampaio de Almeida Ribeiro, Rita Barradas Barata, ICV 2020 Group, Adriana Ilha da Silva, Alberto Novaes Ramos, Ana Paula França, Andrea de Nazaré Marvão Oliveira, Antonio Fernando Boing, Carla Magda Allan Santos Domingues, Consuelo Silva de Oliveira, Ethel Leonor Noia Maciel, Ione Aquemi Guibu, Isabelle Ribeiro Barbosa Mirabal, Jaqueline Caracas Barbosa, Jaqueline Costa Lima, José Cássio de Moraes, Karin Regina Luhm, Karlla Antonieta Amorim Caetano, Luisa Helena de Oliveira Lima, Maria Bernadete de Cerqueira Antunes, Maria da Gloria Teixeira, Maria Denise de Castro Teixeira, Maria Fernanda de Sousa Oliveira Borges, Rejane Christine de Sousa Queiroz, Ricardo Queiroz Gurgel, Rita Barradas Barata, Roberta Nogueira Calandrini de Azevedo, Sandra Maria do Valle Leone de Oliveira, Sheila Araújo Teles, Silvana Granado Nogueira da Gama, Sotero Serrate Mengue, Taynãna César Simões, Valdir Nascimento, Wildo Navegantes de Araújo

**Affiliations:** 1 Faculdade de Ciências Médicas da Santa Casa de São Paulo, Departamento de Saúde Coletiva, São Paulo, SP, Brazil; 2 Centro de Vigilância Epidemiológica “Prof. Alexandre Vranjac”, Coordenadoria de Controle de Doenças, São Paulo, SP, Brazil; 3 Coordenadoria de Controle de Doenças, Secretaria de Estado da Saúde de São Paulo, São Paulo, SP, Brazil; Universidade Federal do Espírito Santo, Vitória, ES, Brazil; Universidade Federal do Ceará, Departamento de Saúde Comunitária, Fortaleza, CE, Brazil; Faculdade Ciências Médicas Santa Casa de São Paulo, São Paulo, SP, Brazil; Secretaria de Estado da Saúde do Amapá, Macapá, AP, Brazil; Universidade Federal de Santa Catarina, SC, Brazil; Organização Pan-Americana da Saúde, Brasília, DF, Brazil; Instituto Evandro Chagas, Belém, PA, Brazil; Faculdade de Ciências Médicas Santa Casa de São Paulo, Departamento de Saúde Coletiva, São Paulo, SP, Brazil; Universidade Federal de Mato Grosso, Cuiabá, MT, Brazil; Universidade Federal do Paraná, Curitiba, PR, Brazil; Universidade Federal de Goiás, Goiânia, GO, Brazil; Universidade Federal do Piauí, Teresina, PI, Brazil; Universidade de Pernambuco, Faculdade de Ciências Médicas, Recife, PE, Brazil; Instituto de Saúde Coletiva, Universidade Federal da Bahia, Salvador, BA, Brazil; Secretaria de Estado da Saúde de Alagoas, Maceió, AL, Brazil; Universidade Federal do Acre, Rio Branco, AC, Brazil; Universidade Federal do Maranhão, Departamento de Saúde Pública, São Luís, MA, Brazil; Universidade Federal de Sergipe, Aracaju, SE, Brazil; Secretaria Municipal de Saúde, Boa Vista, RR, Brazil; Fundação Oswaldo Cruz, Mato Grosso do Sul, Campo Grande, MS, Brazil; Fundação Oswaldo Cruz, Escola Nacional de Saúde Pública Sergio Arouca, Rio de Janeiro, RJ, Brazil; Universidade Federal do Rio Grande do Sul, Porto Alegre, RS, Brazil; Fundação Oswaldo Cruz, Instituto de Pesquisa René Rachou, Belo Horizonte, MG, Brazil; Secretaria de Desenvolvimento Ambiental de Rondônia, Porto Velho, RO, Brazil; Universidade de Brasília, Brasília, DF, Brazil

**Keywords:** Encuestas Epidemiológicas, Vacuna contra el Sarampión-Parotiditis-Rubéola, Niño, Cobertura de Vacunación, Vacilación a la Vacunación, Health Surveys, Measles-Mumps-Rubella Vaccine, Child, Vaccination Coverage, Vaccination Hesitancy

## Abstract

**Objective:**

To estimate measles-mumps-rubella vaccination coverage, delay and loss to follow-up in children up to 24 months old living in Brazilian cities.

**Methods:**

Surveys and questionnaires with a retrospective cohort of live births in 2017-2018, analyzing vaccination coverage and sociodemographic data of children and families, based on vaccination card records and interviews.

**Results:**

Valid coverage of first dose was 90.0% (95%CI 88.9;91.0) and 81.1% for the second dose (95%CI 79.8;82.4). Delay for both doses was 23.2% (95%CI 21.9;24.5) and loss to follow-up was 10.8% (95%CI 9.9;11.8). Socioeconomic stratum A had the lowest vaccination coverage and the higher the child’s birth order, the lower the vaccination coverage for the second dose. Children whose mothers had 13 to 15 years of education had higher vaccination coverage.

**Conclusion:**

Coverage did not meet the recommended target. Differentiated strategies to resolve difficulties in access, misinformation, and vaccination hesitancy will help improve vaccination coverage.

## Study contributions

**Main results:**

Coverage of measles-mumps-rubella vaccine first and second doses was below 95%. In most cities, coverage of the first dose was below 80%, falling to between 70% and 80% for the second dose. Dose losses and delays were found in children under 1 year old.

**Implications for services:**

Low coverage levels and missed opportunities increase the risk of reemergence of measles and the occurrence of outbreaks and epidemics. Health services need to adopt active tracing strategies, reminders, visits and active involvement with achieving targets.

**Perspectives:**

Managers of immunization programs at the three levels of government need to promote immunization actions, operationally reorganize services and constantly monitor indicators for vaccine-preventable disease control.

## RESUMEN

**Objetivo:**

Estimar la cobertura de vacunación, retraso y la pérdida en el seguimiento de la vacuna triple vírica en niños de hasta 24 meses residentes en ciudades brasileñas.

**Métodos:**

Encuestas y cuestionarios en una cohorte retrospectiva de nacidos vivos en 2017-2018, análisis de la cobertura y ​​datos sociodemográficos de niños y familias, a partir de registros de cartilla de vacunación y entrevistas.

**Resultados:**

La cobertura válida para la primera dosis: 90,0% (IC95% 88,9;91,0); segunda: 81,1% (IC95% 79,8;82,4). El retraso de ambas: 23,2% (IC95% 21,9;24,5); pérdida en el seguimiento: 10,8% (IC95% 9,9;11,8). El estrato A tuvo la cobertura más baja y cuanto mayor el orden de nacimiento, menor la cobertura para la segunda dosis. Niños con madres que tienían entre 13 y 15 ãnos de escolatización presentaron una mayor cobertura.

**Conclusión:**

La cobertura no alcanzó el recomendado, por lo que diferentes estrategias para resolver las dificultades de acceso, la desinformación y las dudas sobre las vacunas mejorarían la cobertura.

## INTRODUCTION 

Vaccination is the main public health strategy for preventing infectious disease transmission, complications and deaths, the cost-effectiveness ratio of which has the greatest impact on global indicators.^1-4^ The measles vaccine was introduced in Brazil in the 1960s, becoming effective throughout the country with the creation of the National Immunization Program (*Programa Nacional de Imunizações* - PNI), in 1973. From 1992 onwards, the measles, mumps and rubella (MMR) vaccine was gradually incorporated, providing protection against these diseases.^5-7^

Due to the high transmissibility of the measles virus, vaccination coverage needs to be high, in order to reduce transmission chain. The World Health Organization recommends that countries achieve 95% coverage for the measles-mumps-rubella (MMR) vaccine, however, since 2014, Brazil has not achieved the recommendation for the full vaccination schedule.^8,9^ Although the Region of the Americas was declared free of measles in 2016,^10,11^ a measles outbreak occurred in Venezuela in 2017, which went beyond its borders and spread to Colombia, Argentina, Chile, Ecuador and Peru. In 2018, measles reemerged in Brazil, in the Northern region of the country, and in 2019, in São Paulo, with high incidence among children under 5 years old, demonstrating the existence of susceptible (unvaccinated) children.^12^

The Brazilian PNI is considered to be a reference program globally.^13,14^ However, data reported in the literature show a substantial drop in vaccination coverage in Brazil, mainly for Bacillus Calmette-Guérin (BCG), inactivated poliovirus vaccine and MMR.^7,8,15^

In 2017, the Ministry of Health indicated reasons why measles vaccination coverage has fallen, including: the mistaken perception that the virus is no longer in circulation and, therefore, vaccination is unnecessary; the increase in anti-vaccine movements at national and international levels; and incompleteness of the vaccination schedule, justified by a feeling of protection by only taking a single dose.^11^ Public data made available by the Ministry of Health show that vaccination coverage has decreased over the years, and that it fell to approximately 65% ​​due to the COVID-19 pandemic.^16,17^

Given the reduction in coverage, especially with regard to Brazil’s basic vaccination schedule, there is a need to identify the influence of the social and economic context on the population’s behavior, especially with regard to vaccination adherence. Discussions on the topic and data analysis play a fundamental role in preventing and detecting changes in the population’s individual or collective health patterns. The objective of this study was therefore to estimate MMR vaccination coverage, delay and loss to follow-up, among children up to 24 months old living in Brazilian cities. 

## METHODS

This study is a population-based household survey looking specifically at MMR vaccination, forming part of the National Vaccination Coverage Survey 2020,^18^ based on a probabilistic sample of the cohort of children born alive between 2017 and 2018, in 26 state capitals, the Federal District and 12 cities with more than 100,000 inhabitants located outside the metropolitan regions of the state capital cities (Imperatriz-Maranhão, Caruaru-Pernambuco, Sobral-Ceará, Vitória da Conquista-Bahia, Sete Lagoas-Minas Gerais, Petrópolis-Rio de Janeiro, Campinas-São Paulo, Joinville-Santa Catarina, Londrina-Paraná, Rio Grande-Rio Grande do Sul, Rio Verde-Goiás and Rondonópolis-Mato Grosso). 

The study’s field data collection period extended from September 2020 to March 2022. To calculate the sample size, a formula^18^ was used which took into consideration: DEFF (design effect due to the use of clusters of census tracts) of 1.4; a hypothetical population of 1 million live births; estimated 70% coverage prevalence, with a 5% estimation error; and a z score of 1.96, for a 95% confidence interval. This resulted in 452 children per survey. As such, the researchers calculated a predicted sample of 37,836 live births from the 2017 and 2018 cohorts.

The sample of children was divided into socioeconomic strata, within the census tracts in which they lived (strata A-D), classified based on information from the 2010 Demographic Census,^18^ which used data on average income of heads of household, proportion of literate heads of household and proportion of heads of household with income greater than or equal to 20 minimum wages. The census tracts were grouped by means of cluster analysis, using Euclidean distance and adjusting the results to define four strata, containing at least the minimum number of children born in 2017 or 2018 necessary to reach the expected sample size, ensuring that each stratum had approximately the same number of children and compensated for the number of losses over the course of the study. Stratum A refers to the high-income socioeconomic group; B = medium high income; C = medium low income; and D = low income.^18^

The data for calculating coverage, losses, delays and vaccination before one year old were obtained from each child’s vaccination card, while the intermediate variables were obtained by means of a structured questionnaire with questions related to the sociodemographic variables specified below. 

Maternal variables:

1. Levels of schooling – years (≤ 8; 9-12; 13-15; ≥ 16; unable to answer; not informed);
2. Age group – years (≤ 20; 20-34; ≥ 35);
3. Race/skin color (White, Black, mixed race, Asian, Indigenous and not informed);
4. Has a job (yes/no);
5. Marital status/Partner (yes/no);
6. Number of children;
7. Grandmother lives in the household (yes/no).

Child variables:

1. Sex (female/male);
2. Birth order (first, second, third, fourth or above);
3. Race/skin color;
4. Attends daycare (yes/no).

Family variables:

1. Level of consumer goods (A-B; C-D; not informed);
2. Monthly family income – BRL (≤ 1000; 1001-3000; 3001-8000; ≥ 8001; not informed);
3. Bolsa *Família* income transfer program beneficiary (yes, no, not informed).

Variables for assessing coverage:

1. Administered doses (first dose; second dose);
2. Valid doses (first dose; second dose);
3. On-time doses (first dose; second dose);
4. Delayed doses (first dose; second dose; both doses);
5. Lost to follow-up;
6. Dose administered before 1 year old.

Considering the objectives of this study, administered MMR doses and administration dates were verified. When calculating the first and second doses, we took into account both MMR doses and MMRV doses (measles, mumps, rubella and varicella). 

Administered doses are those recorded on vaccination cards. Valid doses are those administered according to the age defined by the national schedule and with minimum intervals between them. On-time doses are those administered exactly during the period specified by the schedule and with minimum intervals between them. Coverage was calculated considering the relationship between the number of doses administered and the number of children with an assessed vaccination card, multiplied by 100.

In the case of the first dose of MMR or MMRV vaccines, doses administered after 365 days of life or more are considered valid, and those administered between 365 and 394 days of life (from 12 to 13 months) are considered on-time. In the case of the second dose, vaccines administered at least 30 days after the first dose was administered are valid; while on-time vaccines are those administered between 452 and 486 days of life (around 15 to 16 months).

The first dose was considered to be delayed if it was administered 30 days or more after 12 months; and the second dose was delayed if it was administered after 487 days or more (30-35 days after 15 months). Based on these definitions, the following indicators were compiled:

- Indicator of delay: delay in the period recommended for administering the first dose and delay in the second dose.
- Indicator of loss to follow-up: proportion of children who received the first dose and did not return for the second dose.
- Coverage of the first valid dose: relationship between the number of first valid doses and the number of vaccinated children, multiplied by 100.
- Coverage of the second valid dose: relationship between the number of second valid doses and the number of vaccinated children, multiplied by 100.
- Proportion of administered doses in children under 1 year old.

Each indicator was assessed independently, without using a classification bar, taking the values ​​of the indicator itself.

With the aim of incorporating different aspects of a vaccination program and based on the variables available in the vaccination survey, a standardized performance indicator was developed, which aims to evaluate the efficiency of an immunization program, by municipality. 

Standardized Performance Indicator: the five indicators (delay, loss to follow-up, coverage and administered doses in children under 1 year of age) were normalized using the z score (z = value of the indicator in each municipality-mean value/standard deviation). The indicator was obtained by summing the z-scores of each indicator for each municipality. The higher the z-score, the better the performance.

The software used for statistical analysis was the Statistical Package for the Social Sciences (SPSS version 13), applying the definitions of weights, strata and clusters to calculate coverage estimates and loss percentages. 95% confidence intervals (95%CI) were calculated for all estimates, considering the complex sampling plan.

### Ethical considerations 

The National Vaccination Coverage Survey 2020 was approved by the Research Ethics Committee of the *Instituto de Saúde Coletiva da Universidade Federal da Bahia*, as per Opinion No. 3.366.818, on June 4, 2019, and Certificate of Submission for Ethical Appraisal (*Certificado de Apresentação de Apreciação* Ética - CAAE) No. 4306919.5.0000.5030; and by the Research Ethics Committee of the *Irmandade da Santa Casa de São Paulo*, as per Opinion No. 4.380.019, on November 4, 2020, and CAAE No. 39412020.0.0000.5479. 

## RESULTS

Interviews were carried out in relation to 37,836 of the expected initial sample of 40,050 individuals, with 5.53% losses and refusals; 35 children were excluded because they were outside the age cohort established for the survey, therefore, 37,801 children were assessed.^18^ Of the total number of children assessed, 34,338 (90.84%) received at least one dose of MMR vaccine. MMR vaccine coverage at 12 months taking all state capitals, municipalities and the Federal District included in the survey was 90.9% (95%CI 89.9;91.9), considering administered doses. This indicator hardly changed in relation to valid doses, for which coverage was 90.0% (95%CI 88.9;91.0). However, using the on-time criterion caused coverage to drop drastically to 52.3% (95%CI 50.6;53.9) (Table 1). As seen in Table 1, the coverage indicators for the second dose show coverage to be reduced by approximately 8.0% for both valid and on-time doses: respectively, to 81.1% (95%CI 79.8;82.4) and 40.2% (95%CI 38.7;41.7).

**Table 1:** Estimated MMR and MMRV vaccination coverage indicators (%) and 95% confidence intervals (95%CI) for state capitals, other cities and Federal District together, Brazil, 2020-2021

Indicator	Total children vaccinated	% (95%CI)	
**Administered doses**			
First dose	34,338	90.9 (89.9;91.9)	
Second dose	31,091	82.2 (80.9;83.5)	
**Valid doses**			
First dose	33,908	90.0 (88.9;91.0)	
Second dose	30,710	81.1 (79.8;82.4)	
**On-time doses**			
First dose	18,899	52.3 (50.6;53.9)	
Second dose	14,618	40.2 (38.7;41.7)	
Delayed first dose	15,009	37.7 (36.2;39.2)	
Delayed second dose	15,788	49.4 (47.3;50.6)	
Both doses delayed	9,514	23.2 (21.9;24.5)	
Loss to follow-up	3,628	10.8 (9.9;11.8)	
Dose administered before 1 year old	1,633	4.6 (3.9;5.3)	

The first dose was delayed for just under 40.0% of children, and the second dose was delayed for around half of them. One tenth of children showed loss of coverage between the first and second dose. The vaccine was administered before 12 months of age to approximately 5.0% of children (Table 1).

Table 2 shows, in bold type, cities for which the difference in coverage between the first and second administered doses was greater than 10.0%. The results are sorted in descending order, based on coverage including the second dose. There was great heterogeneity in coverage among Brazilian cities, with only two cities having coverage estimates above 90.0%; 20 cities, between 89.0% and 80.0%; 14 cities, between 79.0% and 70.0%; and two between 69.0% and 60.0%. Teresina had the highest coverage for the second booster (91.1%, 95%CI 86.1;94.4), followed by Curitiba (90.5%, 95%CI 86.1;93.6). At the other extreme are Florianópolis (72.0%, 95%CI 66.0;77.3), Vitória (69.6%, 95%CI 59.3;78.2), Natal (67.6% , 95%CI 54.1;78.8) and Rio Grande (65.4%, 95%CI 53.2;75.9). 

**Table 2:** Estimated MMR and MMRV vaccination coverage (%) and 95% confidence intervals (95%CI), by administered doses for state capitals, other cities and Federal District, Brazil, 2020-2021

City	n	First administered dose		Second administered dose		First and second doses
		Freq.	Vaccination coverage % (95%CI)	Freq.	Vaccination coverage % (95%CI)	
Teresina	899	852	96.9 (93.8;98.4)	793	91.1 (86.1;94.4)	5.8
Curitiba	1,192	1,123	94.1 (91.1;96.2)	1,073	90.5 (86.1;93.6)	3.6
Joinville	460	452	96.7 (93.4;98.3)	412	89.5 (83.7;93.4)	7.2
Sete Lagoas	451	440	96.5 (88.0;99.0)	406	89.1 (83.0;93.2)	7.4
Brasília	1,809	1,649	90.2 (87.0;92.7)	1,585	88.6 (85.7;90.9)	1.6
Londrina	1,818	442	96.7 (93.1;98.4)	402	87.3 (78.2;92.9)	9.4
Salvador	455	1,677	93.6 (90.8;95.6)	1,562	86.9 (82.5;90.4)	6.7
Caruaru	462	449	96.6 (92.9;98.4)	414	86.7 (79.1;91.9)	9.9
Campinas	1,383	1,654	85.5 (71.1;93.4)	1,547	86.1 (80.4;90.3)	-0.6
Porto Alegre	451	1,261	89 (83.4;92.9)	1,182	85.8 (80.7;89.8)	3.2
Porto Velho	1,774	419	94.3 (90.8;96.5)	380	85.7 (79.9;90.1)	8.6
Sobral	465	390	91.4 (77.7;97.0)	337	84.4 (68.2;93.1)	7
Belo Horizonte	1,863	1,667	88.9 (83.5;92.7)	1,573	83.7 (78.7;87.7)	5.2
Boa Vista	1,689	368	91 (81.8;95.8)	311	83.4 (76.5;88.6)	7.6
Recife	468	1,554	91.9 (85.2;95.7)	1,361	83.2 (77.4;87.7)	8.7
São Paulo	1,539	1,447	92.9 (90.2;94.9)	1,334	83.1 (79.8;86.0)	9.8
Petrópolis	395	447	88.2 (71.1;95.8)	417	82.1 (69.7;90.1)	6.1
Goiânia	1,811	1,612	87.9 (82.1;91.9)	1,477	81.7 (74.2;87.5)	6.2
Cuiabá	814	748	91.2 (86.9;94.2)	652	81.4 (74.8;86.6)	9.8
Rio Verde	444	408	91.4 (86.4;94.7)	363	81 (72.1;87.5)	10.4
Macapá	878	792	92.2 (88.0;95.0)	700	80.8 (77.4;83.8)	11.4
Aracaju	1,826	807	87.1 (79.0;92.4)	732	80.2 (71.2;86.9)	6.9
Manaus	900	1,732	94.7 (92.1;96.5)	1,478	79.7 (75.0;93.8)	15
São Luís	854	774	88.8 (79.0;94.4)	678	79.4 (72.0;85.2)	9.4
Palmas	465	390	85 (81.7;87.9)	345	77.6 (70.4;83.5)	7.4
Belém	1,612	1,119	87.8 (81.4;92.2)	1,017	77.3 (66.8;85.2)	10.5
Imperatriz	453	439	93.1 (88.5;96.0)	382	76.8 (69.2;83.0)	16.3
Campo Grande	929	1,139	89.2 (86.3;91.5)	1,008	76.3 (70.2;81.6)	12.9
Fortaleza	904	1,443	87.3 (81.8;91.3)	1,291	75.9 (66.7;83.1)	11.4
Maceió	1,281	801	82.9 (69.0;91.4)	723	75.8 (65.2;84.0)	7.1
Rio Branco	1,218	406	90.5 (87.8;92.7)	347	75.7 (69.9;80.7)	14.8
João Pessoa	451	830	88.8 (82.9;92.9)	730	75.7 (68.8;81.5)	13.1
Rondonópolis	449	374	85.1 (74.9;91.6)	324	75.5 (67.6;82.0)	9.6
Vitória da Conquista	455	353	84.7 (69.6;93.1)	317	74.1 (63.4;82.6)	10.6
Rio de Janeiro	788	1,535	83.2 (78.1;87.3)	1,344	72.7 (67.6;77.2)	10.5
Florianópolis	739	658	84.5 (78.1;89.3)	582	72 (66.0;77.3)	12.5
Vitória	452	711	86.7 (76.2;92.9)	649	69.6 (59.3;78.2)	17.1
Natal	1,820	597	83.5 (75.9;89.1)	521	67.6 (54.1;78.8)	15.9
Rio Grande	685	379	76.9 (65.4;85.4)	342	65.4 (53.2;75.9)	11.5

In Table 3, the cities are arranged in descending order, according to the standardized performance value, with the city of Sete Lagoas showing the best assessment against the set of indicators. In turn, the city of Natal had the poorest performance. Furthermore, the behavior between the state capitals and cities of the Brazilian regions is heterogeneous, and no regional pattern was found.

**Table 3:** MMR and MMRV vaccination performance indicators (%), for state capitals, other cities and Federal District, Brazil, 2020-2021

City	Loss	Delayed	Vaccination coverage < 1 year	Vaccination coverage D1	Vaccination coverage D2	Standardized performance
Sete Lagoas	7.9	13.2	2.7	96.5	89.1	1.826
Teresina	6.1	30.8	0.4	96.9	91.1	1.517
Joinville	8.3	16.6	4.9	96.7	89.5	1.407
Curitiba	5.1	23.9	3.9	94.1	90.5	1.305
Brasília	3.8	20.5	3.1	90.2	88.6	1.304
Belo Horizonte	6.6	11.3	2.8	88.9	83.7	1.254
Londrina	10.2	21.1	3.2	96.7	87.3	1.160
Sobral	7.9	15.1	3.0	91.4	84.4	1.146
Salvador	8.1	23.7	2.7	93.6	86.9	1.034
Caruaru	10.3	27.8	3.7	96.6	86.7	0.735
Porto Velho	10.5	23.9	3.6	94.3	85.7	0.722
Porto Alegre	5.8	28.5	1.9	89.0	85.8	0.703
Campinas	6.5	21.3	4.4	85.5	86.1	0.496
Petrópolis	7.1	25.3	2.5	88.2	82.1	0.471
Aracaju	9.3	23.2	1.0	87.1	80.2	0.411
São Paulo	11.0	19.3	6.7	92.9	83.1	0.328
Recife	9.9	21.9	6.2	91.9	83.2	0.281
Rio Verde	12.8	28.0	1.0	91.4	81.0	0.243
Goiânia	8.1	27.4	3.3	87.9	81.7	0.155
Maceió	9.9	17.5	2.3	82.9	75.8	0.010
Vitoria da Conquista	12.6	16.4	1.7	84.7	74.1	-0.038
Cuiabá	11.4	32.1	2.9	91.2	81.4	-0.069
Fortaleza	13.9	22.5	3.7	87.3	75.9	-0.397
Boa Vista	10.1	26.2	9.8	91.0	83.4	-0.405
Macapá	13.2	32.6	6.4	92.2	80.8	-0.585
Rio de Janeiro	13.1	22.0	2.4	83.2	72.7	-0.591
Joao Pessoa	15.4	32.3	1.7	88.8	75.7	-0.650
Campo Grande	15.6	26.8	4.4	89.2	76.3	-0.660
Imperatriz	17.5	32.2	3.5	93.1	76.8	-0.673
Rio Branco	17.2	36.5	0.9	90.5	75.7	-0.785
Belém	12.5	30.2	5.6	87.8	77.3	-0.786
Palmas	10.1	28.5	6.8	85.0	77.6	-0.828
Rondonópolis	11.9	31.9	4.7	85.1	75.5	-0.975
Manaus	16.8	31.3	9.2	94.7	79.7	-0.998
São Luís	14.2	32.7	7.7	88.8	79.4	-1.107
Florianópolis	15.2	23.8	6.3	84.5	72.0	-1.229
Rio Grande	15.6	22.0	2.0	76.9	65.4	-1.485
Vitória	20.1	17.2	12.4	86.7	69.6	-1.954
Natal	19.6	35.9	5.4	83.5	67.6	-2.294

Table 4 shows the coverage, delay and loss to follow-up indicators according to socioeconomic stratum. In relation to valid first dose coverage, stratum A had lower coverage than that of strata C and D. With regard to two valid doses, only stratum A had lower coverage than stratum C. Stratum D had the biggest drop in coverage between the first dose (90.6%; 95%CI 89.0;92.0) and the second dose (81.6%; 95%CI 79.3;82,8). When looking at delay in administering the two doses of the vaccine, all strata show similar behavior, but it is noteworthy that the estimate per point was higher in stratum A (27.5%, 95%CI 23.9;31.5 ), and lower in stratum D. 

**Table 4:** MMR and MMRV vaccination indicators (%) and 95% confidence intervals (95%CI), by socioeconomic strata, for state capitals, other cities and Federal District, Brazil, 2020-2021

Strata	First valid dose		Two valid doses		Both doses delayed		Loss to follow-up	
	Freq.	Vaccination coverage % (95%CI)	Freq.	Vaccination coverage % (95%CI)	Freq.	% (95%CI)	Freq.	% (95%CI)
A	7,276	84.7 (80.6;88.0)	6590	76.0 (70.4;80.7)	2,181	27.5 (23.9;31.5)	805	12.4 (9.2;16.6)
B	8,389	89.1 (85.6;91.8)	7,593	81.4 (77.5;84.7)	2,371	25.6 (21.1;29.5)	909	9.3 (7.3;11.8)
C	9,095	91.8 (90.4;93.1)	8,283	83.9 (82.1;85.6)	2,512	24.3 (22.4;26.2)	917	9.6 (8.2;11.2)
D	9,148	90.6 (89.0;92.0)	8,244	81.1 (79.3;82.8)	2,450	21.3 (19.6;23.2)	997	11.3 (10.0;12.7)

Assessment of the children’s birth order showed that, as birth order increased, the estimated coverage for the two valid doses was lower. Children with mothers who had 13 to 15 years of schooling had the highest coverage, with 83.5% (95%CI 81.9;85.2) for two valid doses, which is higher coverage than that for children with mothers with up to 8 years of schooling. No other variable related to the characteristics of the mother, family and child was associated with coverage (Table 5). 

**Table 5:** Vaccination coverage (%) and 95% confidence intervals (95%CI) for both doses of MMR and MMRV vaccines, according to maternal and child information and family socioeconomic characteristics, in state capitals, other cities and Federal District, Brazil, 2020-2021

Maternal and child information and socioeconomic characteristics	Two valid doses	
	Freq.	Vaccination coverage % (95%CI)
Maternal race/skin color		
White	12,459	82.2 (80.1;84.1)	
Black	3,512	83.5 (80.4;86.1)	
Mixed race	13,639	79.9 (78.0;81.7)	
Asian	314	87.4 (75.8;93.9)	
Indigenous	100	83.3 (70.6;91.2)	
Not informed	686	63.8 (55.3;71.6)	
**Maternal age group (years)**		
< 20	703	81.5 (73.2;87.6)	
20-34	17,440	80.8 (79.3;82.3)	
> 35	12,434	81.5 (79.3;83.6)	
**Partner**		
Yes	23,156	81.7 (80.1;83.2)	
No	6,729	81.4 (79.1;83.5)	
**Maternal job**		
Yes	13,506	82.0 (80.3;83.6)	
No	16,539	81.2 (79.3;83.0)	
**Maternal schooling (years)**		
≤ 8	2,629	77.7 (73.5;81.4)	
9-12	4,402	79.4 (76.5;82.1)	
13-15	12,549	83.6 (81.9;85.2)	
≥ 16	10,376	81.0 (78.2;83.6)	
Unable to answer	754	65.2 (57.1;72.5)	
Not informed	887	64.6 (56.7;71.9)	
**Grandmother living in same household**		
Yes	8,036	80.1 (77.8;82.3)	
No	22,629	81.5 (80.0;82.9)	
**Child’s sex**		
Male	15,787	80.8 (79.1;82.3)	
Female	14,923	81.4 (79.6;83.2)	
**Birth order**		
First	15,125	83.6 (81.8;85.1)	
Second	9,806	80.5 (78.0;82.8)	
Third	3,690	78.9 (75.5;82.1)	
Fourth or above	2,068	72.7 (67.5;77.3)	
**Level of consumer goods**		
A-B	8,969	81.5 (78.5;84.2)	
C-D	20,854	81.8 (80.4;83.1)	
Not informed	887	64.6 (56.7;71.9)	
**Monthly family income (BRL)**			
≤ 1000	6,988	79.6 (77.5;81.5)	
1001-3000	10,336	83.1 (81.3;84.9)	
3001-8000	6,167	84.0 (81.2;86.4)	
≥ 8001	3,837	83.3 (78.8;87.1)	
Not informed	3,382	74.5 (69.0;79.3)	
*Bolsa* *Família* **benefit**		
Yes	8,342	82.7 (80.9;84.4)	
No	22,266	80.5 (78.9;82.1)	
Not informed	102	84.8 (74.2;91.5)	

## DISCUSSION

The PNI target is to achieve coverage greater than 95% for the two doses of the MMR vaccine.^19^ However, the state capitals and cities assessed in this study did not meet the target for both doses, as also found by other epidemiological studies that assessed measles vaccination in Brazil between 2020 and 2021.^17,20,21^

In 2018, with the reemergence of measles in Brazil, the sustained circulation of the virus and the low vaccination coverage found by this study, Brazil recorded 9,325 cases and 12 deaths, with a greater concentration of cases in the Northern region.^22^ In 2022, with support from the Pan American Health Organization, Brazil began the process of recertification of the elimination of the measles virus. Since then, the epidemiological scenario continues to be monitored in order to provide the necessary indicators.^22^ As such, elimination of the measles virus demands a commitment between government agencies and the population.

Given the percentage of doses considered to be on-time, it can be seen that half of the people eligible for vaccination are receiving vaccines in the non-ideal period, this being a factor that can influence individual and collective protection.^23^ Furthermore, fourteen cities showed a difference greater than 10% between first and second dose coverage, making clear the important role of missed opportunities for booster dose vaccination in gaining an understanding of low coverage rates.

Only the cities of Teresina and Joinville had coverage greater than 95% for the first dose of the MMR vaccine. With the aim of refining understanding of the importance of different vaccination times and opportunities, we assessed the performance indicator related to the MMR vaccination schedule, whereby nine cities showed better performance, although only five of them are state capital cities. Loss to follow-up and delay in administering doses are important factors that determine the final performance of vaccination actions. Detailing these components makes it possible to highlight some distinct patterns, such as that of Teresina, which obtained the highest coverage for the second dose, but not the best performance, as the dose was delayed for 31% of the children; or that of Belo Horizonte, which has a considerable overall performance, but coverage for the first dose below the average of the cities in the survey. At the other extreme of performance are Natal, followed by Vitória and Rio Grande, which had delay indicators below the average of the cities in the survey. This is not about ranking the cities, but pointing out performance weaknesses in each of the components considered.

States such as Acre, Amazonas, Goiás, Mato Grosso, Mato Grosso do Sul, Rio Grande do Sul and Paraná have indigenous peoples living in hard-to-access areas and border populations, which are considered more vulnerable and, therefore, differentiated strategies are needed to reach theses specific populations and obtain high and homogeneous coverage. In turn, other states, such as São Paulo – which has the largest port in Latin America and the largest airport in South America – have a large circulation of people, as well as receiving refugees and returnees, factors that can alter the local epidemiological pattern and facilitate the introduction or reintroduction of diseases.^18,24^

Vaccination coverage surveys carried out around 40 years ago showed reduced coverage in the lowest socioeconomic strata, which was not seen in the following ten years. However, this scenario changed in the 2000s.^20,22^ In this study, it could be seen that, regarding the first valid dose, only stratum A had lower coverage than strata C and D. Considering the second dose, only stratum A had lower coverage than stratum C. However, the children with mothers who had more than 13 years of schooling had the highest coverage. This suggests that mothers with higher education levels can consult more reliable information regarding the importance of vaccination.^25,26^

This study used secondary data, which has some limitations. Sampling restricted to the urban areas of the state capitals, the Federal District and twelve cities in the interior region does not allow coverage estimates to be inferred for the entire country, or even for differences between regions. Difficulties in accessing residents, due to urban insecurity, the COVID-19 pandemic and also the lack of interest in participating, especially among families from higher socioeconomic strata, as already reported in other household surveys, may lead to some selection bias. Although this study incorporated a complex sampling plan, it did not investigate the importance of contextual variables associated with the characteristics of clusters and individual variables and their interactions. To do so, it would be necessary to use a multilevel model, which was not within the scope of this analysis.

The reduction in coverage in Brazil and worldwide is multifactorial and may be related to the complexity of the vaccination schedule, lack of access, lack of correct and reliable information, and continuous changes in information systems. These associated factors often lead to vaccination hesitancy, which also plays an important role in the fall in coverage,^14^ which corroborates the findings of another study,^18^ in which the reasons for not vaccinating were as follows: medical contraindications, difficulties in access, problems in the functioning of the program and vaccination hesitancy.

Therefore, multi-vaccination campaigns in several easily accessible locations (health centers, schools, mobile vaccination services, etc.), assessing the need to administer vaccines considered “backlogged”, have been essential for improving coverage. It is very useful to make the most of the moment to identify the causes of delayed vaccination.

Improving the efficiency of immunization programs, with the adoption of differentiated strategies, is essential for resolving difficulty of access, misinformation and vaccination hesitancy. As such, promoting educational campaigns and governmental and non-governmental, national and international partnerships is essential in order to achieve improvement in coverage, with an emphasis on the quality of health worker training (continuing education), availability of resources (human, financial and material) and improvement of work processes (alignment of work flows, data collection and information systems).
