# Supplementary material for: Vaccination coverage, delay and loss to follow-up of the triple viral vaccine, in live births between 2017 and 2018 in Brazilian cities
Source: Epidemiol Serv Saude. 2025 Jan 10;33(spe2):e20231218. doi: 10.1590/S2237-96222024v33e20231218.especial2.en (PMC11734600; doi:10.1590/S2237-96222024v33e20231218.especial2.en)
Supplement: Translation

# Translation: Cobertura vacinal, atraso e perda de seguimento da vacina tríplice viral, em nascidos entre 2017 e 2018, em cidades brasileiras

**DOI:** 10.1590/S2237-96222024v33e20231218.especial2.pt

**Authors:** Tatiana Lang D’Agostini, Fernanda Florencia Fregnan Zambom, José Cássio de Moraes, Ana Paula França, Jéssica Pires de Camargo, Manoel Carlos Sampaio de Almeida Ribeiro, Rita Barradas Barata, Grupo ICV 2020, Adriana Ilha da Silva, Alberto Novaes Ramos, Ana Paula França, Andrea de Nazaré Marvão Oliveira, Antonio Fernando Boing, Carla Magda Allan Santos Domingues, Consuelo Silva de Oliveira, Ethel Leonor Noia Maciel, Ione Aquemi Guibu, Isabelle Ribeiro Barbosa Mirabal, Jaqueline Caracas Barbosa, Jaqueline Costa Lima, José Cássio de Moraes, Karin Regina Luhm, Karlla Antonieta Amorim Caetano, Luisa Helena de Oliveira Lima, Maria Bernadete de Cerqueira Antunes, Maria da Gloria Teixeira, Maria Denise de Castro Teixeira, Maria Fernanda de Sousa Oliveira Borges, Rejane Christine de Sousa Queiroz, Ricardo Queiroz Gurgel, Rita Barradas Barata, Roberta Nogueira Calandrini de Azevedo, Sandra Maria do Valle Leone de Oliveira, Sheila Araújo Teles, Silvana Granado Nogueira da Gama, Sotero Serrate Mengue, Taynãna César Simões, Valdir Nascimento, Wildo Navegantes de Araújo

**Affiliations:** 1 Faculdade de Ciências Médicas da Santa Casa de São Paulo, Departamento de Saúde Coletiva, São Paulo, SP, Brasil; 2 Centro de Vigilância Epidemiológica “Prof. Alexandre Vranjac”, Coordenadoria de Controle de Doenças, São Paulo, SP, Brasil; 3 Coordenadoria de Controle de Doenças, Secretaria de Estado da Saúde de São Paulo, São Paulo, SP, Brasil; Universidade Federal do Espírito Santo, Vitória, ES, Brasil; Universidade Federal do Ceará, Departamento de Saúde Comunitária, Fortaleza, CE, Brasil; Faculdade Ciências Médicas Santa Casa de São Paulo, São Paulo, SP, Brasil; Secretaria de Estado da Saúde do Amapá, Macapá, AP, Brasil; Universidade Federal de Santa Catarina, SC, Brasil; Organização Pan-Americana da Saúde, Brasília, DF, Brasil; Instituto Evandro Chagas, Belém, PA, Brasil; Faculdade de Ciências Médicas Santa Casa de São Paulo, Departamento de Saúde Coletiva, São Paulo, SP, Brasil; Universidade Federal de Mato Grosso, Cuiabá, MT, Brasil; Universidade Federal do Paraná, Curitiba, PR, Brasil; Universidade Federal de Goiás, Goiânia, GO, Brasil; Universidade Federal do Piauí, Teresina, PI, Brasil; Universidade de Pernambuco, Faculdade de Ciências Médicas, Recife, PE, Brasil; Instituto de Saúde Coletiva, Universidade Federal da Bahia, Salvador, BA, Brasil; Secretaria de Estado da Saúde de Alagoas, Maceió, AL, Brasil; Universidade Federal do Acre, Rio Branco, AC, Brasil; Universidade Federal do Maranhão, Departamento de Saúde Pública, São Luís, MA, Brasil; Universidade Federal de Sergipe, Aracaju, SE, Brasil; Secretaria Municipal de Saúde, Boa Vista, RR, Brasil; Fundação Oswaldo Cruz, Mato Grosso do Sul, Campo Grande, MS, Brasil; Fundação Oswaldo Cruz, Escola Nacional de Saúde Pública Sergio Arouca, Rio de Janeiro, RJ, Brasil; Universidade Federal do Rio Grande do Sul, Porto Alegre, RS, Brasil; Fundação Oswaldo Cruz, Instituto de Pesquisa René Rachou, Belo Horizonte, MG, Brasil; Secretaria de Desenvolvimento Ambiental de Rondônia, Porto Velho, RO, Brasil; Universidade de Brasília, Brasília, DF, Brasil

**Keywords:** Inquéritos Epidemiológicos, Vacina Tríplice Viral, Criança, Cobertura Vacinal, Hesitação Vacinal

## Abstract

**Objetivo:**

Estimar cobertura vacinal, atraso e perda de seguimento da vacina tríplice viral, em crianças até 24 meses residentes em cidades brasileiras.

**Método:**

Inquérito domiciliar baseado em uma coorte retrospectiva de nascidos vivos em 2017-2018, que analisou cobertura e dados sociodemográficos das crianças e famílias, a partir dos registros da caderneta de vacinação e entrevista.

**Resultados:**

A cobertura válida da primeira dose foi de 90,0% (IC_95%_ 88,9;91,0), e da segunda, e 81,1% (IC_95%_ 79,8;82,4). O atraso para ambas as doses foi de 23,2% (IC_95%_ 21,9;24,5) e a perda de seguimento de 10,8% (IC_95%_ 9,9;11,8). O estrato socioeconômico A apresentou menor cobertura e, à medida que a ordem de nascimento aumentava, menor era a cobertura para as duas doses. Crianças de mães com 13 a 15 anos de escolaridade apresentaram maior cobertura.

**Conclusão:**

As coberturas não alcançaram a meta preconizada. Estratégias diferenciadas para dirimir dificuldade de acesso, desinformação e hesitação poderão melhorar a cobertura.

## Contribuições do estudo

**Principais resultados:**

As coberturas da primeira e segunda doses da vacina tríplice viral foram inferiores a 95%. Na maioria das cidades, a cobertura da primeira dose foi inferior a 80%, caindo para 70 a 80%, na segunda. Observaram-se perdas e atrasos de doses em crianças menores de 1 ano.

**Implicações para os serviços:**

Coberturas baixas e oportunidades perdidas aumentam o risco de reintrodução do sarampo e ocorrência de surtos e epidemias. Os serviços devem adotar estratégias de busca ativa, lembretes, visitas e envolvimento ativo com o alcance das metas.

**Perspectivas:**

Os gestores dos programas de imunizações nos três níveis de governo devem fomentar ações de imunização, reorganizar operacionalmente os serviços e monitorar constantemente os indicadores para o controle das doenças imunopreveníveis.

## INTRODUÇÃO 

A vacinação é a principal estratégia de saúde pública para prevenir a transmissão, complicações e óbitos por doenças infectocontagiosas, cuja relação custo-efetividade mais impacta os indicadores mundiais.^1-4^ A vacina contra o sarampo foi introduzida no Brasil na década de 1960, tornando-se efetiva em todo o território com o Programa Nacional de Imunizações (PNI), em 1973. A partir de 1992, foi incorporada gradativamente a vacina tríplice viral, que confere proteção contra sarampo, caxumba e rubéola.^5-7^

Devido à alta transmissibilidade do vírus do sarampo, a cobertura vacinal precisa ser elevada, diminuindo a cadeia de transmissão. A Organização Mundial da Saúde recomenda que os países atinjam 95% de cobertura para a vacina tríplice viral, porém, desde 2014, o Brasil não atinge a recomendação para o esquema completo.^8,9^ Apesar de a região das Américas ter sido declarada livre de sarampo em 2016,^10,11^ ocorreu um surto de sarampo na Venezuela, em 2017, que extrapolou as fronteiras e chegou à Colômbia, à Argentina, ao Chile, ao Equador e ao Peru. Em 2018, a doença foi reintroduzida no território brasileiro, na região Norte, e em 2019, em São Paulo, com incidência elevada em crianças menores de 5 anos, demonstrando a presença de suscetíveis (não vacinados) neste grupo.^12^

O PNI é considerado um programa de referência mundial.^13,14^ Porém, dados da literatura mostram queda substancial da cobertura no Brasil, principalmente para as vacinas contra o bacilo de Calmette-Guérin (BCG), vacina inativada contra poliomielite e tríplice viral.^7,8,15^

Em 2017, o Ministério da Saúde indicou razões pelas quais a cobertura do sarampo declinou, entre elas: a falsa percepção da não circulação do vírus e, portanto, a vacinação seria desnecessária; aumento dos movimentos antivacina nos âmbitos nacional e internacional; e incompletude do esquema vacinal, justificada por uma sensação de proteção com uma única dose.^11^ Dados públicos do Ministério da Saúde mostram que as coberturas vacinais decresceram ao longo dos anos, e a cobertura vacinal caiu para aproximadamente 65% devido à pandemia de covid-19.^16,17^

Frente à redução das coberturas, em especial do calendário básico de vacinação do país, faz-se necessário identificar a influência do contexto social e econômico no comportamento da população, principalmente no que diz respeito à adesão à vacinação. As discussões acerca do tema e a análise de dados possuem um papel fundamental na prevenção e detecção de mudanças nos padrões de saúde individual ou coletiva da população. Assim, o objetivo deste estudo foi estimar a cobertura vacinal, o atraso e a perda de seguimento da vacina tríplice viral, em crianças até 24 meses residentes em cidades brasileiras. 

## MÉTODOS

Este estudo é um inquérito domiciliar de base populacional dirigido à vacinação de tríplice viral, que integra o Inquérito Nacional de Cobertura Vacinal – 2020,^18^ baseado numa amostra probabilística da coorte de crianças nascidas vivas entre 2017 e 2018, em 26 capitais, no Distrito Federal e em 12 municípios com mais de 100 mil habitantes localizados fora das regiões metropolitanas das capitais (Imperatriz-Maranhão, Caruaru-Pernambuco, Sobral-Ceará, Vitória da Conquista-Bahia, Sete Lagoas-Minas Gerais, Petrópolis-Rio de Janeiro, Campinas-São Paulo, Joinville-Santa Catarina, Londrina-Paraná, Rio Grande-Rio Grande do Sul, Rio Verde-Goiás e Rondonópolis-Mato Grosso). 

O período de coleta de campo do estudo se estendeu de setembro de 2020 a março de 2022. Para calcular o tamanho da amostra, utilizou-se uma fórmula^18^ em que se considerou: EDFF (efeito do desenho pela utilização de conglomerados de setores censitários) de 1,4; população hipotética de 1 milhão de nascidos vivos; prevalência estimada de cobertura de 70%, com erro de estimativa de 5%; e z de 1,96, para intervalo de confiança de 95%. Chegou-se ao número de 452 crianças por inquérito. Com isso, os pesquisadores obtiveram uma amostragem prevista de 37.836 nascidos vivos das coortes de 2017 e 2018.

A amostra de crianças foi obtida em estratos socioeconômicos, dos setores censitários de residência (estratos A-D), classificados com base nas informações do Censo Demográfico de 2010,^18^ que utilizaram os dados de renda média dos responsáveis pelo domicílio, proporção de responsáveis alfabetizados e proporção de responsáveis com renda maior ou igual a 20 salários mínimos. Os setores foram agrupados por meio da análise de *cluster*, utilizando-se distância euclidiana e ajustando-se os resultados para a definição de quatro estratos, com pelo menos o número mínimo de crianças nascidas em 2017 ou 2018 necessário para atingir o tamanho da amostra prevista, garantindo que cada estrato apresentasse aproximadamente o mesmo número de crianças e compensasse o número de perdas ao longo do estudo. O estrato A refere-se ao grupo socioeconômico de renda alta; o B, de renda média alta; o C, de média baixa; e o D, de renda baixa.^18^

Os dados para o cálculo das coberturas, perdas, atrasos e vacinação antes do primeiro ano foram obtidos da caderneta de vacinação de cada criança, e as variáveis intermediárias, por questionário estruturado com perguntas relacionadas às variáveis sociodemográficas especificadas a seguir. 

Variáveis da mãe:

1. Níveis de escolaridade – anos (≤ 8; 9-12; 13-15; ≥ 16; não sabe; sem informação);
2. Faixa etária –anos (≤ 20; 20-34; ≥ 35);
3. Raça/cor da pele (branca, preta, parda, amarela, indígena e sem informação);
4. Trabalho (sim/não);
5. Estado civil/Companheiro (sim/não);
6. Número de filhos;
7. Avó mora junto (sim/não).

Variáveis da criança:

1. Gênero (feminino/masculino);
2. Ordem de nascimento (primeiro, segundo, terceiro, quarto ou mais);
3. Raça/cor da pele;
4. Se frequenta creche (sim/não).

Variáveis da família:

1. Nível de bens de consumo (A-B; C-D; sem informação);
2. Renda familiar mensal – R$ (≤ 1.000; 1.001-3.000; 3.001-8.000; ≥ 8.001; sem informação);
3. Bolsa Família (sim, não, sem informação).

Variáveis para avaliação da cobertura:

1. Doses aplicadas (primeira dose; segunda dose);
2. Doses válidas (primeira dose; segunda dose);
3. Doses oportunas (primeira dose; segunda dose);
4. Atraso (na primeira dose; na segunda; nas duas doses);
5. Perda do seguimento;
6. Dose aplicada antes de 1 ano.

Considerando-se os objetivos deste estudo, foram observadas as doses aplicadas e as datas para a tríplice viral. Para o cálculo da primeira e da segunda dose, foram consideradas tanto as doses das vacinas tríplice viral, quanto da tetraviral (contra sarampo, caxumba, rubéola e varicela). 

Doses aplicadas são aquelas registradas na caderneta de vacinas. Doses válidas são aquelas aplicadas conforme a idade prevista no calendário nacional e com intervalos mínimos entre elas. Doses oportunas são aquelas aplicadas exatamente no período previsto no calendário e com intervalos mínimos entre elas. O cálculo da cobertura considerou a relação entre o número de doses aplicadas e o número de crianças com a caderneta de vacinação avaliada, multiplicado por 100.

Para a primeira dose das vacinas tríplice ou tetraviral, são consideradas válidas as doses aplicadas com 365 dias de vida ou mais, e oportunas aquelas aplicadas entre 365 e 394 dias de vida (dos 12 aos 13 meses). Para a segunda dose, são válidas as vacinas aplicadas pelo menos 30 dias após a aplicação da primeira dose; e as oportunas, aquelas aplicadas entre 452 e 486 dias de vida (em torno dos 15 a 16 meses).

A primeira dose foi considerada atrasada se aplicada 30 dias ou mais após os 12 meses; e a segunda dose, se aplicada com 487 dias ou mais (30-35 dias após os 15 meses). Com base nestas definições, foram construídos os seguintes indicadores:

- Indicador de atraso: atraso no período proposto para a aplicação da primeira dose e atraso da segunda dose.
- Indicador de perda de seguimento: proporção de crianças que receberam a primeira dose e não retornaram para a aplicação da segunda dose.
- Cobertura da primeira dose válida: relação entre o número de primeiras doses válidas e o número de crianças vacinadas multiplicado por 100.
- Cobertura da segunda dose válida: relação entre o número de segundas doses válidas e o número de crianças vacinadas multiplicado por 100.
- Proporção das doses aplicadas em menores de 1 ano.

Cada indicador foi avaliado de maneira independente, sem utilização de barema de classificação, considerando-se os valores do indicador em si.

Com o intuito de incorporar diferentes aspectos de um programa de vacinação e com base nas variáveis disponíveis no inquérito vacinal, foi construído o indicador de desempenho padronizado, que tem por objetivo avaliar a eficiência de um programa de imunização, segundo município. 

Indicador de Desempenho Padronizado: os cinco indicadores (atraso, perda de seguimento, coberturas e doses aplicadas em menores de 1 ano) foram normalizados pelo escore z (z = valor do indicador em cada município-valor médio/desvio-padrão). O indicador foi obtido pela somatória dos escores z de cada indicador para cada município. Quanto maior o escore z, melhor o desempenho.

O *software* utilizado para as análises estatísticas foi o Statistical Package for the Social Sciences (SPSS versão 13), aplicando-se as definições de pesos, estratos e conglomerados para os cálculos das estimativas de cobertura e porcentagens de perda. Para todas as estimativas, foram calculados os intervalos de confiança de 95% (IC_95%_), considerando-se o plano complexo de amostragem.

### Considerações éticas

O Inquérito Nacional de Cobertura Vacinal 2020 foi aprovado pelos Comitês de Ética em Pesquisa com Seres Humanos do Instituto de Saúde Coletiva da Universidade Federal da Bahia, sob parecer n^o^ 3.366.818, em 4 de junho de 2019, com Certificado de Apresentação de Apreciação Ética (CAAE) 4306919.5.0000.5030; e da Irmandade da Santa Casa de São Paulo, sob parecer n^o^ 4.380.019, em 4 de novembro de 2020, com CAAE 39412020.0.0000.5479. 

## RESULTADOS

Da amostra inicial prevista de 40.050 indivíduos, foram entrevistados 37.836, com 5,53% de perdas e recusas; 35 crianças foram excluídas por estarem fora da coorte de idade estabelecida no inquérito, portanto, foram avaliadas 37.801 crianças.^18^ Do total de crianças avaliadas, 34.338 (90,84%) receberam ao menos uma dose de vacina tríplice viral. A cobertura aos 12 meses para a vacina tríplice viral de todas as capitais, municípios e Distrito Federal do inquérito foi de 90,9% (IC_95%_ 89,9;91,9%), considerando-se as doses aplicadas. Pouca alteração ocorre neste indicador ao consideramos as doses válidas, sendo a cobertura de 90,0% (IC_95%_ 88,9;91,0%). A utilização do critério de oportunidade faz, no entanto, a cobertura cair drasticamente para os 52,3% (IC_95%_ 50,6;53,9) (Tabela 1). Como observado na Tabela 1, os indicadores de cobertura, considerando-se a segunda dose, reduzem em aproximadamente 8,0% a cobertura tanto para doses válidas quanto para as oportunas: respectivamente, para 81,1% (IC_95%_ 79,8;82,4%) e 40,2% (IC_95%_ 38,7;41,7).

**Tabela 1:** Indicadores (%) e intervalos de confiança de 95% (IC_95_) de cobertura vacinal estimada para tríplice e tetraviral no conjunto das capitais, cidades e Distrito Federal, 2020-2021

Indicador	Total de crianças vacinadas	% (IC_95_ **%**)	
**Doses aplicadas**			
Primeira dose	34.338	90,9 (89,9;91,9)	
Segunda dose	31.091	82,2 (80,9;83,5)	
**Doses válidas**			
Primeira dose	33.908	90,0 (88,9;91,0)	
Segunda dose	30.710	81,1 (79,8;82,4)	
**Doses oportunas**			
Primeira dose	18.899	52,3 (50,6;53,9)	
Segunda dose	14.618	40,2 (38,7;41,7)	
Atraso na primeira dose	15.009	37,7 (36,2;39,2)	
Atraso na segunda dose	15.788	49,4 (47,3;50,6)	
Atraso nas duas doses	9.514	23,2 (21,9;24,5)	
Perda de seguimento	3.628	10,8 (9,9;11,8)	
Dose aplicada antes de 1 ano	1.633	4,6 (3,9;5,3)	

Houve atraso da primeira dose em pouco menos de 40,0% das crianças, e da segunda, em cerca da metade. Um décimo das crianças mostrou perda de cobertura entre a primeira e a segunda dose. A vacina foi aplicada antes dos 12 meses de idade em aproximadamente 5,0% das crianças (Tabela 1).

A Tabela 2 mostra, em negrito, as cidades cuja diferença entre as coberturas de primeira e segunda dose aplicadas foi maior que 10,0%. Os resultados estão ordenados de forma decrescente, com base na cobertura incluindo a segunda dose. Houve uma grande heterogeneidade da cobertura entre as cidades brasileiras, sendo que apenas duas cidades apresentaram estimativas de cobertura acima de 90,0%; 20 cidades, entre 89,0 e 80,0%; 14 cidades, entre 79,0% e 70,0%; e duas entre 69,0% e 60,0%. Teresina apresentou a maior cobertura para segundo reforço (91,1%, IC_95%_ 86,1;94,4%), seguida por Curitiba (90,5%, IC_95%_ 86,1;93,6). No outro extremo estão Florianópolis (72,0%, IC_95%_ 66,0;77,3%), Vitória (69,6%, IC_95%_ 59,3;78,2%), Natal (67,6%, IC_95%_ 54,1;78,8%) e Rio Grande (65,4%, IC_95%_ 53,2;75,9). 

**Tabela 2:** Cobertura vacinal estimada (%) e intervalos de confiança de 95% (IC_95%_) de tríplice viral e tetraviral segundo doses aplicadas por capitais, cidades e Distrito Federal, 2020-2021

Cidade	n	Primeira dose aplicada		Segunda dose aplicada		Primeira e segunda doses
		Freq.	Cobertura vacinal % (IC_95%_)	Freq.	Cobertura vacinal % (IC_95%_)	
Teresina	899	852	96,9 (93,8;98,4)	793	91,1 (86,1;94,4)	5,8
Curitiba	1.192	1.123	94,1 (91,1;96,2)	1.073	90,5 (86,1;93,6)	3,6
Joinville	460	452	96,7 (93,4;98,3)	412	89,5 (83,7;93,4)	7,2
Sete Lagoas	451	440	96,5 (88,0;99,0)	406	89,1 (83,0;93,2)	7,4
Brasília	1.809	1.649	90,2 (87,0;92,7)	1.585	88,6 (85,7;90,9)	1,6
Londrina	1.818	442	96,7 (93,1;98,4)	402	87,3 (78,2;92,9)	9,4
Salvador	455	1.677	93,6 (90,8;95,6)	1.562	86,9 (82,5;90,4)	6,7
Caruaru	462	449	96,6 (92,9;98,4)	414	86,7 (79,1;91,9)	9,9
Campinas	1.383	1.654	85,5 (71,1;93,4)	1.547	86,1 (80,4;90,3)	-0,6
Porto Alegre	451	1.261	89 (83,4;92,9)	1.182	85,8 (80,7;89,8)	3,2
Porto Velho	1.774	419	94,3 (90,8;96,5)	380	85,7 (79,9;90,1)	8,6
Sobral	465	390	91,4 (77,7;97,0)	337	84,4 (68,2;93,1)	7
Belo Horizonte	1.863	1.667	88,9 (83,5;92,7)	1.573	83,7 (78,7;87,7)	5,2
Boa Vista	1.689	368	91 (81,8;95,8)	311	83,4 (76,5;88,6)	7,6
Recife	468	1.554	91,9 (85,2;95,7)	1.361	83,2 (77,4;87,7)	8,7
São Paulo	1.539	1.447	92,9 (90,2;94,9)	1.334	83,1 (79,8;86,0)	9,8
Petrópolis	395	447	88,2 (71,1;95,8)	417	82,1 (69,7;90,1)	6,1
Goiânia	1.811	1.612	87,9 (82,1;91,9)	1.477	81,7 (74,2;87,5)	6,2
Cuiabá	814	748	91,2 (86,9;94,2)	652	81,4 (74,8;86,6)	9,8
Rio Verde	444	408	91,4 (86,4;94,7)	363	81 (72,1;87,5)	10,4
Macapá	878	792	92,2 (88,0;95,0)	700	80,8 (77,4;83,8)	11,4
Aracaju	1.826	807	87,1 (79,0;92,4)	732	80,2 (71,2;86,9)	6,9
Manaus	900	1.732	94,7 (92,1;96,5)	1.478	79,7 (75,0;93,8)	15
São Luís	854	774	88,8 (79,0;94,4)	678	79,4 (72,0;85,2)	9,4
Palmas	465	390	85 (81,7;87,9)	345	77,6 (70,4;83,5)	7,4
Belém	1.612	1.119	87,8 (81,4;92,2)	1.017	77,3 (66,8;85,2)	10,5
Imperatriz	453	439	93,1 (88,5;96,0)	382	76,8 (69,2;83,0)	16,3
Campo Grande	929	1.139	89,2 (86,3;91,5)	1.008	76,3 (70,2;81,6)	12,9
Fortaleza	904	1.443	87,3 (81,8;91,3)	1.291	75,9 (66,7;83,1)	11,4
Maceió	1.281	801	82,9 (69,0;91,4)	723	75,8 (65,2;84,0)	7,1
Rio Branco	1.218	406	90,5 (87,8;92,7)	347	75,7 (69,9;80,7)	14,8
João Pessoa	451	830	88,8 (82,9;92,9)	730	75,7 (68,8;81,5)	13,1
Rondonópolis	449	374	85,1 (74,9;91,6)	324	75,5 (67,6;82,0)	9,6
Vitória da Conquista	455	353	84,7 (69,6;93,1)	317	74,1 (63,4;82,6)	10,6
Rio de Janeiro	788	1.535	83,2 (78,1;87,3)	1.344	72,7 (67,6;77,2)	10,5
Florianópolis	739	658	84,5 (78,1;89,3)	582	72 (66,0;77,3)	12,5
Vitória	452	711	86,7 (76,2;92,9)	649	69,6 (59,3;78,2)	17,1
Natal	1.820	597	83,5 (75,9;89,1)	521	67,6 (54,1;78,8)	15,9
Rio Grande	685	379	76,9 (65,4;85,4)	342	65,4 (53,2;75,9)	11,5

Na Tabela 3, as cidades estão dispostas em ordem decrescente, de acordo com o valor do desempenho padronizado, sendo que o município de Sete Lagoas apresentou a melhor avaliação frente ao conjunto de indicadores. Por seu turno, a cidade de Natal obteve o menor desempenho. Ademais, o comportamento entre as capitais e cidades das regiões do Brasil é heterogêneo, não expressando um padrão regional.

**Tabela 3:** Indicadores de desempenho (%) para as vacinas tríplice e tetraviral por capitais, cidades e Distrito Federal, 2020-2021

Cidade	Perda	Atraso	Cobertura vacinal < 1 ano	Cobertura vacinal D1	Cobertura vacinal D2	Desempenho padronizado
Sete Lagoas	7,9	13,2	2,7	96,5	89,1	1,826
Teresina	6,1	30,8	0,4	96,9	91,1	1,517
Joinville	8,3	16,6	4,9	96,7	89,5	1,407
Curitiba	5,1	23,9	3,9	94,1	90,5	1,305
Brasília	3,8	20,5	3,1	90,2	88,6	1,304
Belo Horizonte	6,6	11,3	2,8	88,9	83,7	1,254
Londrina	10,2	21,1	3,2	96,7	87,3	1,160
Sobral	7,9	15,1	3,0	91,4	84,4	1,146
Salvador	8,1	23,7	2,7	93,6	86,9	1,034
Caruaru	10,3	27,8	3,7	96,6	86,7	0,735
Porto Velho	10,5	23,9	3,6	94,3	85,7	0,722
Porto Alegre	5,8	28,5	1,9	89,0	85,8	0,703
Campinas	6,5	21,3	4,4	85,5	86,1	0,496
Petrópolis	7,1	25,3	2,5	88,2	82,1	0,471
Aracaju	9,3	23,2	1,0	87,1	80,2	0,411
São Paulo	11,0	19,3	6,7	92,9	83,1	0,328
Recife	9,9	21,9	6,2	91,9	83,2	0,281
Rio Verde	12,8	28,0	1,0	91,4	81,0	0,243
Goiânia	8,1	27,4	3,3	87,9	81,7	0,155
Maceió	9,9	17,5	2,3	82,9	75,8	0,010
Vitoria da Conquista	12,6	16,4	1,7	84,7	74,1	-0,038
Cuiabá	11,4	32,1	2,9	91,2	81,4	-0,069
Fortaleza	13,9	22,5	3,7	87,3	75,9	-0,397
Boa Vista	10,1	26,2	9,8	91,0	83,4	-0,405
Macapá	13,2	32,6	6,4	92,2	80,8	-0,585
Rio de Janeiro	13,1	22,0	2,4	83,2	72,7	-0,591
Joao Pessoa	15,4	32,3	1,7	88,8	75,7	-0,650
Campo Grande	15,6	26,8	4,4	89,2	76,3	-0,660
Imperatriz	17,5	32,2	3,5	93,1	76,8	-0,673
Rio Branco	17,2	36,5	0,9	90,5	75,7	-0,785
Belém	12,5	30,2	5,6	87,8	77,3	-0,786
Palmas	10,1	28,5	6,8	85,0	77,6	-0,828
Rondonópolis	11,9	31,9	4,7	85,1	75,5	-0,975
Manaus	16,8	31,3	9,2	94,7	79,7	-0,998
São Luís	14,2	32,7	7,7	88,8	79,4	-1,107
Florianópolis	15,2	23,8	6,3	84,5	72,0	-1,229
Rio Grande	15,6	22,0	2,0	76,9	65,4	-1,485
Vitória	20,1	17,2	12,4	86,7	69,6	-1,954
Natal	19,6	35,9	5,4	83,5	67,6	-2,294

A Tabela 4 apresenta os indicadores de cobertura, atraso e perda de seguimento segundo estrato socioeconômico. Em relação à cobertura para uma dose válida, o estrato A apresentou cobertura menor que a dos estratos C e D. Por sua vez, a cobertura para duas doses válidas do estrato A apresentou percentual mais baixo apenas que a cobertura estimada para o estrato C. Já o estrato D ostentou a perda mais importante entre os níveis de cobertura da primeira dose (90,6%; IC_95%_ 89,0;92,0) e para a segunda dose (81,6%; IC_95%_ 79,3;82,8). Considerando-se o atraso na aplicação das duas doses da vacina, todos os estratos apresentam um comportamento próximo, mas vale destacar que a estimativa por ponto foi maior no estrato A (27,5%, IC_95%_ 23,9;31,5), e menor no estrato D. 

**Tabela 4:** Indicadores (%) e intervalos de confiança de 95% (IC_95_), segundo o estrato socioeconômico para as vacinas tríplice e tetraviral por capitais, cidades e Distrito Federal, 2020-2021

Estrato	Primeira dose válida		Duas doses válidas		Atraso nas duas doses		Perda de seguimento	
	Freq.	Cobertura vacinal % (IC_95%_)	Freq.	Cobertura vacinal % (IC_95%_)	Freq.	% (IC_95%_)	Freq.	% (IC_95%_)
A	7.276	84,7 (80,6;88,0)	6.590	76,0 (70,4;80,7)	2.181	27,5 (23,9;31,5)	805	12,4 (9,2;16,6)
B	8.389	89,1 (85,6;91,8)	7.593	81,4 (77,5;84,7)	2.371	25,6 (21,1;29,5)	909	9,3 (7,3;11,8)
C	9.095	91,8 (90,4;93,1)	8.283	83,9 (82,1;85,6)	2.512	24,3 (22,4;26,2)	917	9,6 (8,2;11,2)
D	9.148	90,6 (89,0;92,0)	8.244	81,1 (79,3;82,8)	2.450	21,3 (19,6;23,2)	997	11,3 (10,0;12,7)

A avaliação da ordem de nascimento da criança mostrou que, à medida que a ordem de nascimento aumentava, menor foi a cobertura estimada para as duas doses válidas. As crianças com mães que têm de 13 a 15 anos de escolaridade apresentaram a maior cobertura, com 83,5% (IC_95%_ 81,9;85,2) para duas doses válidas, sendo esta uma cobertura maior que a das crianças com mães com até 8 anos de escolaridade. Nenhuma outra variável relacionada a característica da mãe, da família e da criança se mostrou associada à cobertura (Tabela 5). 

**Tabela 5:** Cobertura vacinal (%) e intervalos de confiança de 95% (IC_95%_) para as duas doses das vacinas tríplice e tetraviral, segundo informações da mãe e da criança e características socioeconômicas da família, nas capitais, municípios e Distrito Federal, 2020-2021

Informações da mãe e da criança e características socioeconômicas	Duas doses válidas	
	Freq.	Cobertura vacinal % (IC_95%_)
Raça/cor da pele da mãe		
Branca	12.459	82,2 (80,1;84,1)	
Preta	3.512	83,5 (80,4;86,1)	
Parda	13.639	79,9 (78,0;81,7)	
Amarela	314	87,4 (75,8;93,9)	
Indígena	100	83,3 (70,6;91,2)	
Sem informação	686	63,8 (55,3;71,6)	
**Faixa etária mãe (anos)**		
< 20	703	81,5 (73,2;87,6)	
20-34	17.440	80,8 (79,3;82,3)	
> 35	12.434	81,5 (79,3;83,6)	
**Companheiro**		
Sim	23.156	81,7 (80,1;83,2)	
Não	6.729	81,4 (79,1;83,5)	
**Mãe trabalha**		
Sim	13.506	82,0 (80,3;83,6)	
Não	16.539	81,2 (79,3;83,0)	
**Escolaridade da mãe (anos)**		
≤ 8	2.629	77,7 (73,5;81,4)	
9-12	4.402	79,4 (76,5;82,1)	
13-15	12.549	83,6 (81,9;85,2)	
≥ 16	10.376	81,0 (78,2;83,6)	
Não sabe	754	65,2 (57,1;72,5)	
Sem informação	887	64,6 (56,7;71,9)	
**Avó mora junto**		
Sim	8.036	80,1 (77,8;82,3)	
Não	22.629	81,5 (80,0;82,9)	
**Sexo da criança**		
Masculino	15.787	80,8 (79,1;82,3)	
Feminino	14.923	81,4 (79,6;83,2)	
**Ordem de nascimento**		
Primeiro	15.125	83,6 (81,8;85,1)	
Segundo	9.806	80,5 (78,0;82,8)	
Terceiro	3.690	78,9 (75,5;82,1)	
Quarto ou mais	2.068	72,7 (67,5;77,3)	
**Nível de bens de consumo**		
A-B	8.969	81,5 (78,5;84,2)	
C-D	20.854	81,8 (80,4;83,1)	
Sem informação	887	64,6 (56,7;71,9)	
**Renda familiar mensal (R$)**			
≤ 1.000	6.988	79,6 (77,5;81,5)	
1.001-3.000	10.336	83,1 (81,3;84,9)	
3.001-8.000	6.167	84,0 (81,2;86,4)	
≥ 8.001	3.837	83,3 (78,8;87,1)	
Sem informação	3.382	74,5 (69,0;79,3)	
**Recebe Bolsa Família**		
Sim	8.342	82,7 (80,9;84,4)	
Não	22.266	80,5 (78,9;82,1)	
Sem informação	102	84,8 (74,2;91,5)	

## DISCUSSÃO

O PNI tem como meta atingir cobertura superior a 95% para as duas doses da vacina tríplice viral.^19^ No entanto, as capitais e municípios avaliados nesse estudo não alcançaram a meta para as duas doses, assim como observado em outros estudos epidemiológicos que avaliaram a vacinação do sarampo no Brasil entre 2020 e 2021.^17,20,21^

Em 2018, com a reintrodução do sarampo no território brasileiro, a circulação sustentada do vírus e as baixas coberturas observadas neste estudo, o Brasil registrou 9.325 casos e 12 óbitos, com uma maior concentração de casos na região Norte.^22^ Em 2022, o Brasil, com apoio da Organização Pan-Americana da Saúde, iniciou o processo de recertificação da eliminação do vírus do sarampo; desde então, continua o monitoramento do cenário epidemiológico, para cumprimento dos indicadores necessários.^22^ Sendo assim, a eliminação do vírus do sarampo demanda um compromisso entre os órgãos governamentais e a população.

No que diz respeito à porcentagem das doses consideradas oportunas, verifica-se que metade das pessoas elegíveis para vacinação estão recebendo as vacinas no período não ideal, fator esse que pode influenciar na proteção individual e coletiva.^23^ Além disso, 14 municípios apresentaram diferença de mais de 10% entre as coberturas de primeira e segunda dose, ficando evidente o importante papel da perda de oportunidade de vacinação da dose de reforço no entendimento das baixas coberturas.

Apenas Teresina e Joinville apresentaram cobertura superior a 95% para a primeira dose da vacina tríplice viral. Buscando-se refinar a compreensão da importância dos diferentes momentos e oportunidades de vacinação, foi avaliado o indicador de desempenho relacionado ao esquema vacinal tríplice viral, sendo que nove cidades apresentaram um melhor desempenho, e entre elas, apenas cinco são capitais. A perda de seguimento e o atraso na administração das doses são importantes fatores que determinam o desempenho final das ações de vacinação. O detalhamento destes componentes permite evidenciar alguns padrões distintos, como o de Teresina, que obteve a maior cobertura para segunda dose, porém não o melhor desempenho, pois 31% das crianças tomaram a dose atrasada; ou o de Belo Horizonte, que possui um desempenho geral considerável, mas uma cobertura para primeira dose abaixo da média das cidades do inquérito. No outro extremo de desempenho, encontram-se Natal, seguido de Vitória e Rio Grande, que apresentaram indicadores de atraso menores que a média das cidades do inquérito. Não se trata de ranquear as cidades, mas apontar as fragilidades de desempenho em cada um dos componentes considerados.

Estados como o Acre, o Amazonas, Goiás, Mato Grosso, Mato Grosso do Sul, Rio Grande do Sul e Paraná possuem povos originários vivendo em áreas de difícil acesso e população de fronteira, que são consideradas mais vulneráveis e, portanto, se fazem necessárias estratégias diferenciadas para atingir este público em específico e se obterem altas e homogêneas coberturas. Por seu turno, outros estados, como São Paulo – que possui o maior porto da América Latina e o maior aeroporto da América do Sul –, apresentam uma grande circulação de pessoas, bem como o acolhimento de refugiados e repatriados, fatores que podem alterar o padrão epidemiológico local e propiciar a introdução ou reintrodução de doenças.^18,24^

Os inquéritos de cobertura realizados há cerca de 40 anos mostravam reduzidas coberturas nos menores estratos socioeconômicos, o que não foi visto nos dez anos seguintes; porém, este cenário se modificou nos anos 2000.^20,22^ Neste estudo, foi possível observar que, para a primeira dose válida, somente o estrato A tem menor cobertura que os estratos C e D. Considerando-se a segunda dose, somente o estrato A tem menor cobertura que o estrato C. Entretanto, as crianças com mães que têm mais de 13 anos de escolaridade apresentaram a maior cobertura. Isso sugere que mães com maior escolaridade possam se basear em informações mais seguras no que diz respeito à importância da vacinação.^25,26^

Este estudo utiliza dados secundários, os quais possuem algumas limitações. A amostragem restrita às áreas urbanas das capitais, do Distrito Federal e de 12 municípios do interior não permite inferir estimativas de cobertura para todo o país, ou mesmo para diferenças entre regiões. As dificuldades de acesso aos moradores, em função da insegurança urbana, da pandemia de covid-19 e, também, do desinteresse em participar, sobretudo entre as famílias dos estratos socioeconômicos mais elevados, como já relatado em outros inquéritos domiciliares, pode acarretar algum viés de seleção. O estudo, embora incorpore o plano complexo de amostragem, não investiga a importância de variáveis contextuais associadas às características dos conglomerados e de variáveis individuais e suas interações. Para tanto, seria necessária a utilização de um modelo multinível, o que não estava no escopo desta análise.

A redução das coberturas no Brasil e no mundo são multifatoriais e podem estar relacionadas à complexidade do calendário vacinal, falta de acesso, falta de informações corretas e confiáveis, e mudanças contínuas dos sistemas de informação. Estes fatores associados, muitas vezes, incorrem na hesitação vacinal, que também possui importante papel na queda das coberturas,^14^ o que corrobora os achados de outro estudo,^18^ no qual os motivos para não vacinação foram os seguintes: contraindicações médicas, dificuldades de acesso, problemas no funcionamento do programa e hesitação vacinal.

Portanto, campanhas de multivacinação em diversos locais de fácil acesso (unidades de saúde, escolas, postos volantes etc.), avaliando-se a necessidade de aplicação de vacinas consideradas “em atraso”, têm sido fundamentais para que haja uma melhora nas coberturas, sendo de grande utilidade aproveitar o momento para identificar as causas do adiamento da vacinação.

A melhora na eficiência dos programas de imunização, com a adoção de estratégias diferenciadas, é fundamental para dirimir a dificuldade de acesso, desinformação e hesitação vacinal. Dessa maneira, a promoção de campanhas educativas e parcerias governamentais e não governamentais, nacionais e internacionais são fundamentais para que se obtenha uma melhora nas coberturas, com ênfase na qualificação da capacitação dos trabalhadores da saúde (educação permanente), disponibilidade de recursos (humanos, financeiros e materiais) e melhoria dos processos de trabalho (alinhamento de fluxos, coleta de dados e sistemas de informação).
